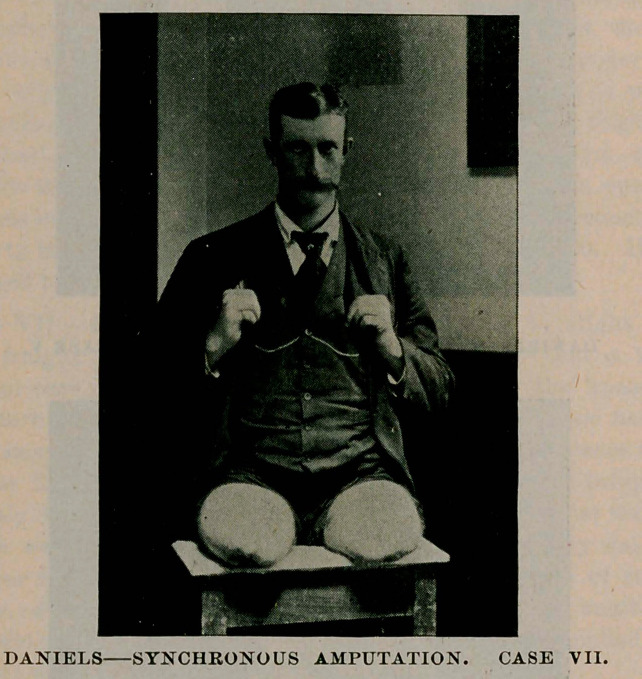# Synchronous Amputations

**Published:** 1895-08

**Authors:** Clayton M. Daniels

**Affiliations:** Buffalo, N. Y.


### SYNCHRONOUS AMPUTATIONS.

By CLAYTON M. DANIELS, M. D., Buffalo, N. Y.

THE mortality in cases of synchronous amputations has materially decreased as surgical science has progressed, until the percentage of fatal results is hardly greater now than in single amputations twenty years ago. Not that every case is suitable for operation, but I believe that more cases can be made so by proper preparatory treatment.

As a basis for my opinion I herewith present histories and illustrations of seven consecutive cases, which embraces my experience with double and triple synchronous amputations, together with some deductions therefrom.

CASE I.—J. Kelly, aged 8½ years. Railway accident; both legs crushed; the left just above the ankle, the right three inches higher

DANIELS—SYNCHRONOUS AMPUTATION. CASE I.

up. He was a strumous, poorly nourished, weakly child. I saw him about forty minutes subsequent to the accident. He had lost considerable blood and was suffering from profound shock; the prospects for operative measures dubious. I stimulated hypodermically very freely, with brandy. Reaction set in promptly. On account of shock and the free stimulation the little patient was anesthetised quickly. Operation occupied twenty-five minutes. Patient placed in bed with hot water bottles about him. He rallied well. The process of repair was very slow; several small pieces of bone were exfoliated from the right tibia. He remained under observation three months, until the stumps were well healed, and now with the aid of his artificial legs he walks without a limp, and is an expert on roller skates and in bicycle riding.

CASE II.—J. Griffin, aged 9½ years. Crush of right foot up to the instep, and of left arm at the upper third; also an epiphyseal fracture

DANIELS—SYNCHRONOUS AMPUTATION. CASE II.

of superior end of right humerus. Patient not suffering from shock to any appreciable extent. Circulation good; body warm; pulse accelerated; otherwise normal. Operation about one hour after injury. Ferguson's modifications of Syme's amputation of right foot. Ordinary flap amputation of left arm. Fracture of humerus reduced. Union by first intention. Recovery and discharged in three weeks.

Case III.—Victor G. Welch, sailor; railway accident; aged 35. Crush of right leg at upper third; left leg at the middle third, and left hand and wrist; fracture of left clavicle, and general contusions. He was knocked down and run over by a car at 12.30 A. M., and not discovered until some time later. He remained in an unconscious state under the car, which had to be raised before he could be extricated from the trucks and wheels. Operation at 3 A. M. Patient had been stimulated to intoxication. Shock not severe. Amputation of right

DANIELS—SYNCHRONOUS AMPUTATION. CASE III.

leg as high as possible without entering knee joint. Left leg four and one-half inches below the knee; and forearm two and one-half inches above the wrist. Fracture of clavicle reduced and patient placed in bed. Operations completed in one hour and fifteen minutes. He rallied well. Upon visiting him four hours later he cheerfully said, "Good morning, doctor; how are you?" and in reference to his serious condition said he had no idea of dying. Union by first intention in all three amputations. He was discharged twenty-five days after injury.

Case IV. H. P., aged 35. Trolley car accident. Both legs crushed at the junction of middle and lower third. Operation an hour and a half after injury. Amputation at nearly the same point. Free stimulation during the operation. As this patient was in the tertiary stage of specific disease his prospects for recovery were in no way promising. Constitutional treatment was continued during the progress of the

DANIELS—SYNCHRONOUS AMPUTATION. CASE IV.

case. He, however, made a good recovery with the exception of some exfoliation of bone from the left tibia.

Case V.—Stanislaus Mackowiak ; railway accident ; aged 23. Complete crush of right leg at knee and of left leg a little lower down. My assistant, Dr. Edward M. Dooley, was called at time of injury, on account of my absence from the city. He amputated the right thigh four inches above the knee, and the left leg at the middle third. On account of the extensive injury to the left leg, which was much worse than appeared on first examination, extensive destruction of the soft parts promptly followed, and traumatic gangrene made rapid progress. Eight days subsequent to the first amputation I re-amputated the left thigh about three and one-half inches above the knee ; so that in this case the man really suffered triple amputation. He made uninterrupted progress. Union by first intention throughout. Here was a case where a man had no desire to live. In fact, he used every effort possible to defeat his recovery, even to the extent of starvation, and for five days after the primary amputation did not taste food, declaring that he would die in spite of us. On the morning of the fifth day, after an earnest inquiry as to his prospects of living, and when I told him that he could not die if he wanted to, he said "If that is the case, please send in my breakfast."

CASE VI. Willie McMahon, aged 12 years. Trolley car accident. Patient suffering severely from shock. Left leg crushed at the knee; right at the ankle. Previous to operating I stimulated hypodermically until his body became warm and proceeded to operate. The pulse at the beginning of operation was 160. Amputation of left thigh three and one-half inches above knee-joint; right leg, at junction of lower and middle third. Free stimulation continued during the operation; equal parts of whisky and ether being used. Operations concluded in about forty minutes. Pulse at that time had dropped to 110. He made a rapid and uninterrupted recovery.

CASE VII.—Owen S., aged 28, while attempting to alight from a moving freight train on February 17, 1895, fell so that a train of thirty-four cars passed over both legs at and below the knees. The temperature was below zero and he laid beside the track for nearly half an hour before found and relief came. The ambulance surgeon could not find pulse or respiration and hesitated about bringing an apparently lifeless body to the hospital. Upon arrival at the hospital there was no improvement and half a pint of whisky was administered per rectum, this being followed by *one-fifth* grain of strychnia sulph. in *divided doses* during the next hour, when another half pint of whisky was given as before, external heat being applied by the use of hot water bottles.

After the first hour, reaction became marked, the patient's condition materially improved and at the end of the second hour he became conscious; an operation thought advisable and I was called. I found the patient quite warm and all external evidences of shock very rapidly passing away. Pulse about 140 per minute. Operation commenced at five minutes past 1 o'clock A. M. Completed in forty-seven minutes. Patient placed in bed and external heat continued. During the operation he was given nine and one-half drachms of whisky hypodermically and one-half drachm of tincture of digitalis. Condition at the end was better than at the beginning of the operation, and at 8 o'clock the same morning he was reading of his injuries in a morning newspaper.

He progressed well for several days, when sloughing of both stumps began, which I believe to have been entirely due to their being frostbitten on the night of injury, although at the time of operation the

DANIELS—SYNCHRONOUS AMPUTATION. CASE V.

DANIELS—SYNCHRONOUS AMPUTATION. CASE VI.

tissues appeared to be in a healthy condition, and to add to the complications was the breaking out of tertiary form of specific disease, seven large ulcers appearing simultaneously. The smallest was larger than a silver dollar and largest about two by three and one-half inches, three of them being upon the thighs which added to the difficulties of dressing the stumps. He was placed upon the "mixed treatment" and has made good progress, all external evidence of the disease having disappeared.

DANIELS—SYNCHRONOUS AMPUTATION. CASE VII.

On account of the sloughing of the stumps and protrusion of the bones, another operation became imperative and five weeks after the injury I re-amputated both thighs, synchronously. He has recovered without a bad symptom and is as cheerful as his pleasant face in the photograph indicates.

DEDUCTION.

In all of these cases I used the precautions known to modern surgery in the way of antiseptic treatment and dressings, each of the limbs being carefully shaved and sterilised, the dressings being of the most approved kind. I use the skin flaps exclusively, and, what I deem as an important point, after the amputation of one extremity, before closing the flaps I amputate the other, in order to watch for any possible hemorrhage that might occur; and after making the second amputation I leave that wound open until

I carefully inspect and close the primary one, being sure to tie or use torsion upon every bleeding point. I believe success is largely due to the care used in preventing hemorrhage. Aseptic catgut ligatures were used in each instance and silk sutures in closing the flaps. I found no use for drainage-tubes, but invariably left a small opening at the lower angle of the wound for drainage purposes, and in those cases where there was not union by first intention I dressed as frequently as the bandage became soiled in the least.

I am a strong advocate of free stimulation immediately before and during the progress of the operation, and believe its value has been fully demonstrated in the series that I have presented. It proves the adage that "heat is life and cold is death." I do not advise operating during the degree of profound shock, neither am I ambitious to operate upon a patient in a moribund condition; but to stimulate freely, and to keep it up even to a degree of semi- or complete intoxication. After the operation warmth should be applied externally by the use of hot bottles, as described. I believe it will save many cases that otherwise would die under the knife.

I prefer hypodermic and rectal stimulation, as the stomach may be inactive, in sympathy with the general condition, and eject its contents. Ordinarily brandy or whisky will suffice, yet if the patient is very low before operating or shows signs of sinking during the operation I add 50 per cent. of clear ether to that given subcutaneously.

As to the time of beginning operation I think it should be done as soon as reaction has set in, not necessarily waiting until it is complete. As soon as moderate warmth of the body occurs, proceed with the work. Although this experience is comparatively limited, yet I am pleased to report seven consecutive recoveries.

315 Jersey Street.

## Figures and Tables

**Figure f1:**
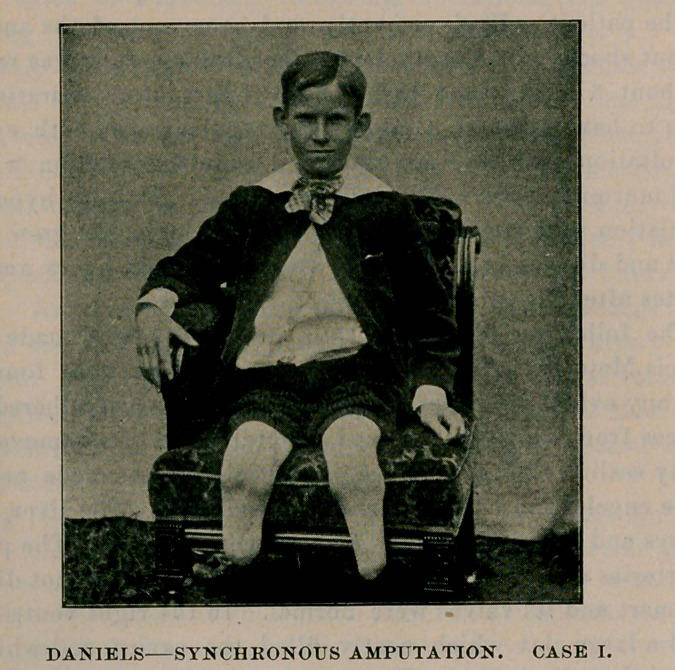


**Figure f2:**
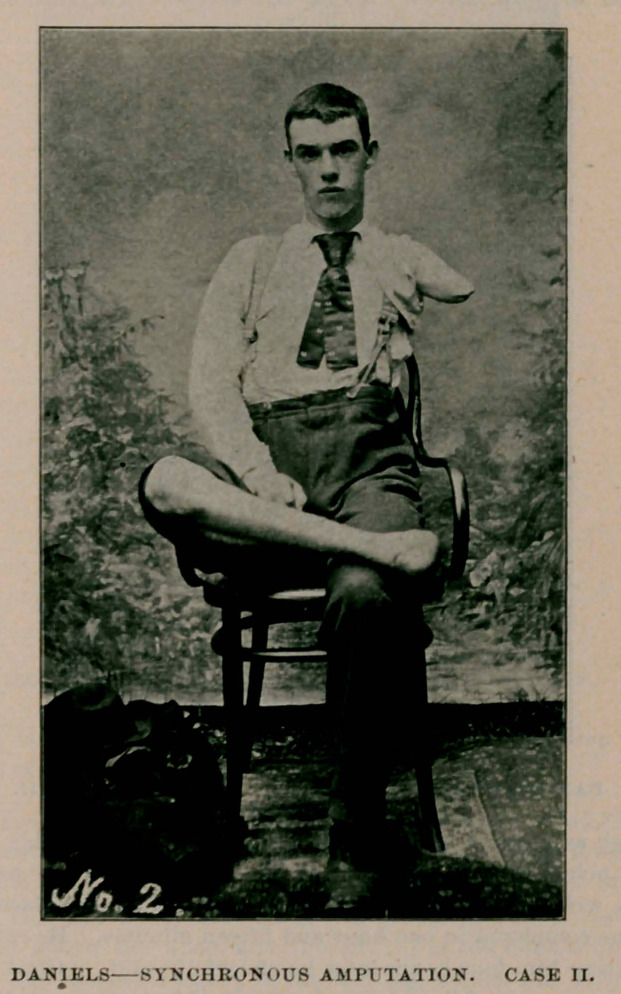


**Figure f3:**
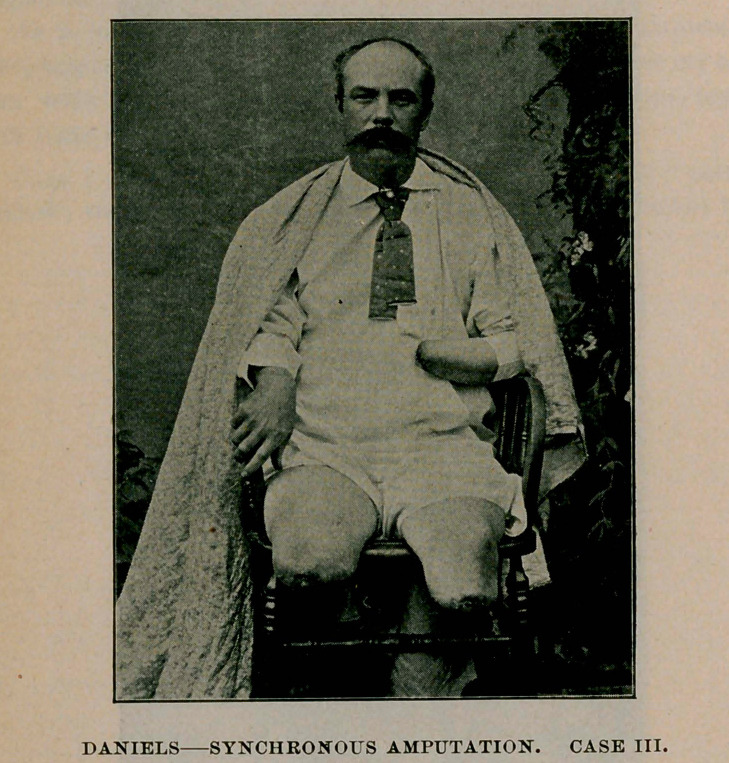


**Figure f4:**
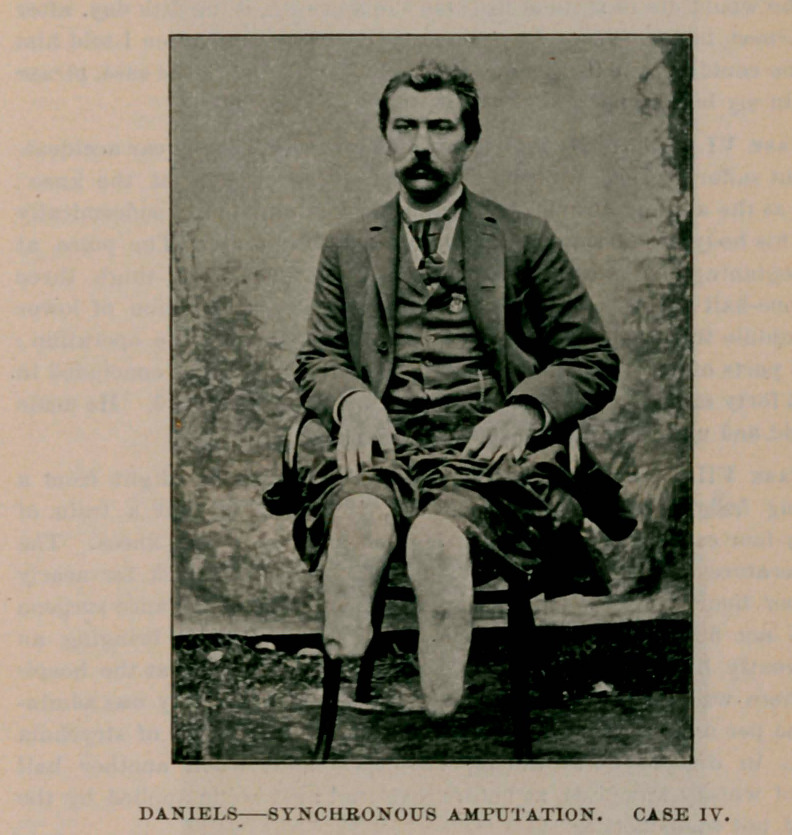


**Figure f5:**
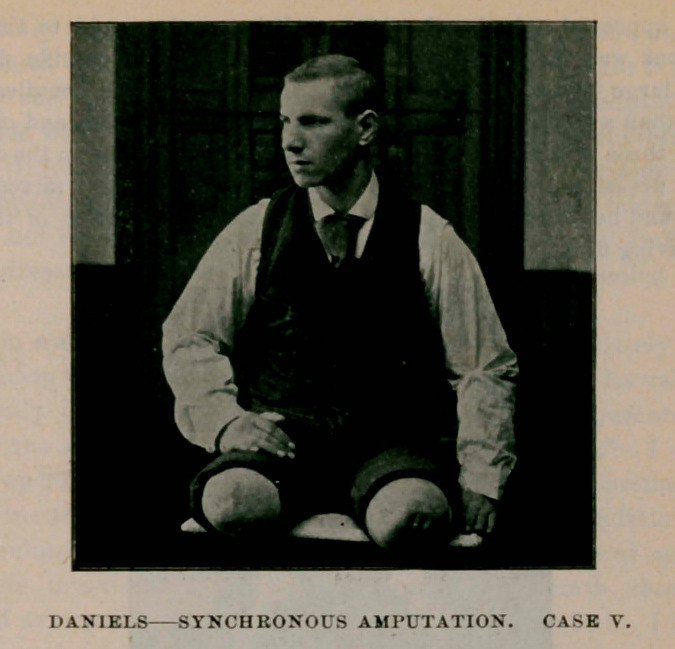


**Figure f6:**
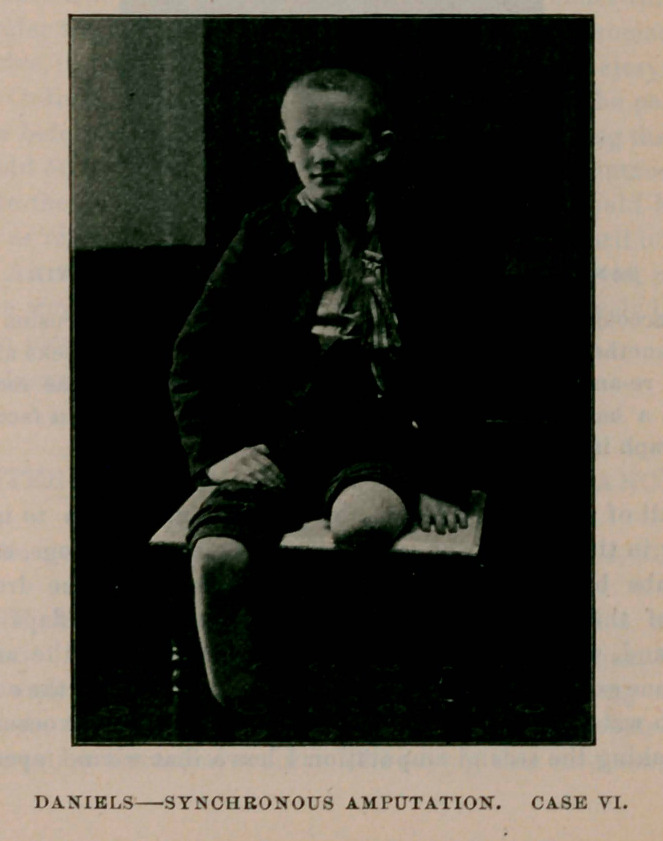


**Figure f7:**